# Targeting Matrix Metalloproteinase-9 to Alleviate T Cell Exhaustion and Improve Sepsis Prognosis

**DOI:** 10.34133/research.0996

**Published:** 2025-11-25

**Authors:** Xuan Wang, Jingyuan Ning, Liang Zhou, Hongru Li, Jinlei Cui, Jiachao Wang, Xiangyang Liang, Jinquan Li, Miao Li, Xue Gao, Wenjian Li, Xing Chen, Fei Yu, Lin Wei, Cuiqing Ma

**Affiliations:** ^1^ Key Laboratory of Immune Mechanism and Intervention on Serious Disease in Hebei Province, Department of Immunology, Hebei Medical University, Shijiazhuang, PR China.; ^2^ Diagnostic Center of Infections, The Second Hospital of Hebei Medical University, Shijiazhuang, PR China.; ^3^ Chinese Academy of Medical Sciences & Peking Union Medical College, Beijing, PR China.; ^4^ Department of Reproductive Medicine, The Second Hospital of Hebei Medical University, Shijiazhuang, PR China.; ^5^ School and Hospital of Stomatology, Hebei Medical University, Shijiazhuang, Hebei, PR China.; ^6^ State Key Laboratory of Agricultural Microbiology, College of Food Science and Technology, Huazhong Agricultural University, Wuhan, Hubei, PR China.; ^7^ Shijiazhuang Heya Biotechnology Co., LTD, Shijiazhuang, PR China.; ^8^ Hebei Key Laboratory of Analysis and Control of Zoonotic Pathogenic Microorganism, College of Life Sciences, Hebei Agricultural University, Baoding, PR China.

## Abstract

Sepsis remains a leading global cause of death, with immune heterogeneity’s molecular mechanisms poorly understood. This study analyzed 1,862 human peripheral blood samples, constructing a molecular interaction disturbance network that first revealed the network biology underlying sepsis immune heterogeneity. We identified 3 sepsis subtypes with marked different prognostic characteristics, with the C1 subtype showing the worst prognosis characterized by severe CD4^+^ T cell exhaustion—validated across 10 independent cohorts. Integrating single-cell transcriptomics from over 450,000 cells, proteomics, and functional validation, we identified monocyte-derived matrix metalloproteinase-9 (MMP9) as a key regulator driving CD4^+^ T cell dysfunction. Mechanistically, MMP9 modulates T cell exhaustion through dual mechanisms: promoting leukocyte-associated immunoglobulin-like receptor-1 (Lair-1) aggregation on T cell membranes, directly inhibiting zeta chain of T cell receptor associated protein kinase 70 (ZAP70) phosphorylation in T cell receptor signaling, while impairing Ca^2+^-release-activated Ca^2+^ channel function and intracellular calcium clearance, causing calcium dysregulation that blocks nuclear factor of activated T cells (NFAT) activation and nuclear translocation. The selective MMP9 inhibitor MMP9-in-1 effectively reversed T cell dysfunction, restored calcium homeostasis and NFAT nuclear translocation, markedly enhanced CD4^+^ T cell interleukin-2 and interferon-γ production, and reduced programmed cell death protein 1 expression. This work establishes a comprehensive translational framework from molecular network disturbances to clinical phenotypes, advancing sepsis immunopathophysiology understanding and providing effective targets for the treatment of sepsis patients.

## Introduction

Sepsis is a life-threatening organ dysfunction caused by a dysregulated host response to infection, representing a significant global health challenge with high morbidity and mortality rates [1,2]. Despite advances in antimicrobial therapy and supportive care, the prognosis of sepsis, particularly in its later stages, remains poor due to persistent immunosuppression [3]. A hallmark of this immunosuppressive state is the impaired number and function of T lymphocytes, especially CD4^+^ T cells, which are critical for coordinating adaptive immune responses [4–11]. Recent studies have identified T cell exhaustion as a defining feature of sepsis, characterized by the sustained expression of inhibitory receptors, such as programmed cell death protein 1 (PD-1); reduced cytokine production; and impaired proliferation [12,13]. However, the immune heterogeneity of septic patients and the upstream molecular mechanisms underlying this dysfunction remain poorly understood [14,15].

With the rapid development of high-throughput sequencing and multi-omics technologies [16,17], researchers have developed various molecular biomarkers and classification methods to improve the diagnosis and treatment of septic patients [18,19]. However, current molecular subtyping tools are often based on single-gene expression profiles or static multigene panels, which tend to overlook the complex interactions between genes within biological pathways [20,21]. It is well established that molecular interaction networks are typically stable in normal tissues but are markedly disrupted in disease states [22]. These network disruptions can more accurately capture the molecular heterogeneity of diseases, providing more precise foundations for patient stratification. Molecular interaction network analysis represents a significant advancement in deciphering disease heterogeneity and optimizing personalized treatment. Unlike traditional gene-expression-based methods, this approach quantifies the extent of changes in gene interactions in disease states, offering deeper insights into the essential characteristics of diseases.

By integrating molecular network disruption analysis, single-cell sequencing technology, proteomics analysis, and functional validation experiments, we unveil a novel immunopathological process in sepsis, in which the infection-induced upregulation of serum matrix metalloproteinase-9 (MMP9) triggers CD4^+^ T cell exhaustion through 2 complementary pathways (T cell receptor [TCR] signal inhibition and calcium homeostasis disruption), and confirm the significant therapeutic effect of targeting MMP9 in restoring T cell function. Previous studies have established that MMP9 suppresses CD8^+^ T cell infiltration and activation and promotes an immunosuppressive environment [23,24], underscoring its critical function in tumor immunosuppression. Our findings extend the role of MMP9 in immunosuppression to the context of infectious diseases. These findings not only provide deeper insights into the immune pathophysiology of sepsis but also establish a critical theoretical foundation and practical framework for implementing clinically relevant combination therapies centered on immunomodulation and anti-infection strategies.

## Results

### Perturbation-based network analysis identifies 3 mechanistically distinct subtypes in sepsis

**Fig. 1.**
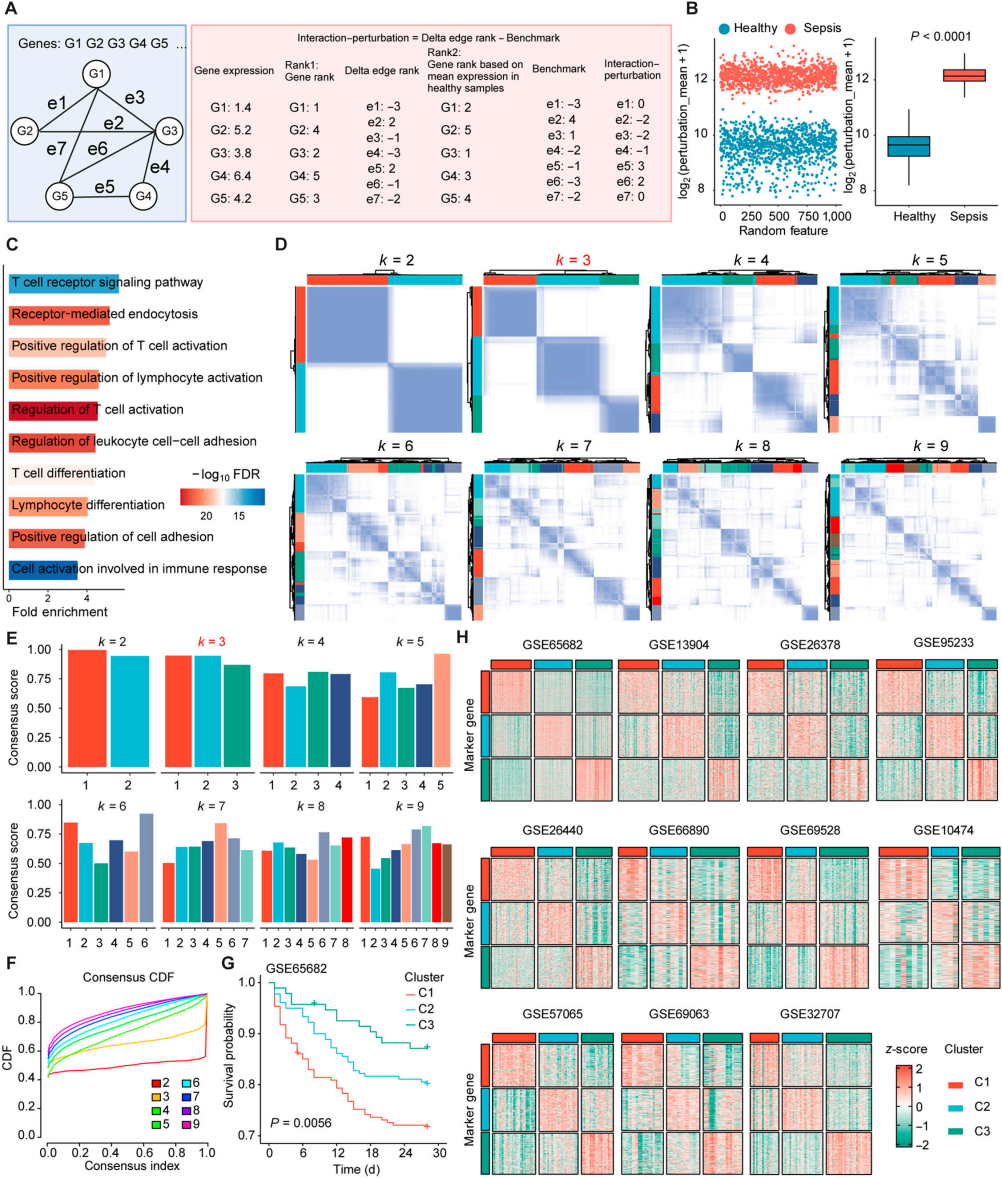
Identification of novel sepsis subtypes using molecular perturbation networks. (A) Schematic diagram of the molecular perturbation network model’s construction. (B) Perturbation degree of each interaction edge between sepsis and healthy control groups. (C) Gene Ontology (GO) enrichment analysis of significantly perturbed node genes between sepsis and healthy control groups. (D) Unsupervised clustering results for *k* values from 1 to 9, showing sample correlations in a heatmap. (E) Consensus scores for *k* values from 1 to 9. (F) Consensus cumulative distribution function (CDF) curves for *k* values from 1 to 9. (G) Survival differences among 3 distinct clusters. (H) Prediction and validation of 3 subtypes identified by the nearest template prediction (NTP) algorithm in external cohorts. FDR, false discovery rate.

We retrieved bulk transcriptome datasets from peripheral blood samples (including both whole blood and peripheral blood mononuclear cells [PBMCs]) from 11 sepsis studies in the Gene Expression Omnibus database, namely, GSE65682, GSE13904, GSE26378, GSE95233, GSE26440, GSE66890, GSE69528, GSE10474, GSE57065, GSE69063, and GSE32707. In total, we collected data on 1,862 samples, comprising 302 healthy controls and 1,560 sepsis samples. Based on previous studies [25], we constructed a molecular interaction disturbance network to explore the molecular mechanisms of sepsis (Fig. 1A). Specifically, we used the bulk transcriptome data from 42 healthy and 760 sepsis peripheral blood samples in the GSE65682 dataset as the training dataset, as this cohort had the largest sample size. The initial rank matrix was obtained from the expression ranks of each gene in individual samples. We then applied subtraction to the rank matrix in the same direction of gene interactions, converting it into a delta rank matrix. Since gene interactions are relatively stable and conserved in healthy samples [26], we used this as the reference benchmark for calculating the disturbance matrix of sepsis samples. The final constructed disturbance network contained 105,598 edges and 4,513 nodes. Previous studies have suggested that biological networks typically exhibit a scale-free distribution [27], which aligns closely with the properties of our constructed network (*R* = −0.853, *P* < 0.0001). Notably, compared to healthy samples, sepsis samples showed stronger disturbance intensity and a broader range of interaction disturbances, indicating significant molecular network alterations in sepsis (Fig. 1B). These results suggest that molecular interaction networks maintain overall stability in healthy samples but undergo significant perturbation in sepsis samples.

To identify the heterogeneity features of sepsis, we retained network disturbance features that significantly differentiate healthy samples from sepsis samples, including 1,155 edges and 770 nodes. Further functional enrichment analysis revealed that these disturbed node genes were primarily enriched in multiple immune-related pathways, including TCR signaling, positive regulation of T cell activation, positive regulation of lymphocyte activation, and leukocyte intercellular adhesion regulation (Fig. 1C). These results suggest that immune system disturbances play a key role in the pathogenesis of sepsis. Based on this disturbance network, we conducted unsupervised clustering analysis. By scanning the stability of subtypes within the range of *k* = 2 to 9, the results showed that *k* = 3 provided the best clarity in the patient-related heatmap (Fig. 1D). Furthermore, when *k* = 3, the consensus scores for each cluster remained above 0.75 (Fig. 1E), and the cumulative distribution function curve also confirmed the high stability of the subtypes at *k* = 3 (Fig. 1F). Kaplan–Meier survival analysis of overall survival (OS) showed significant survival prognosis differences between the 3 clusters, with C1 patients having the worst prognosis, C3 patients having the best prognosis, and C2 patients in an intermediate state (Fig. 1G).

To further validate the reproducibility and universality of these 3 subtypes, we performed validation in 10 additional cohorts. First, differential expression analysis identified the top 300 feature genes for each cluster. We then applied the nearest template prediction (NTP) algorithm to perform external subtype prediction. The results revealed similar gene expression patterns across all validation cohorts (Fig. 1H), confirming the robustness and reproducibility of this subtype classification across platforms and cohorts.

### CD4^+^ T cell exhaustion correlates with adverse outcomes in sepsis patients

**Fig. 2.**
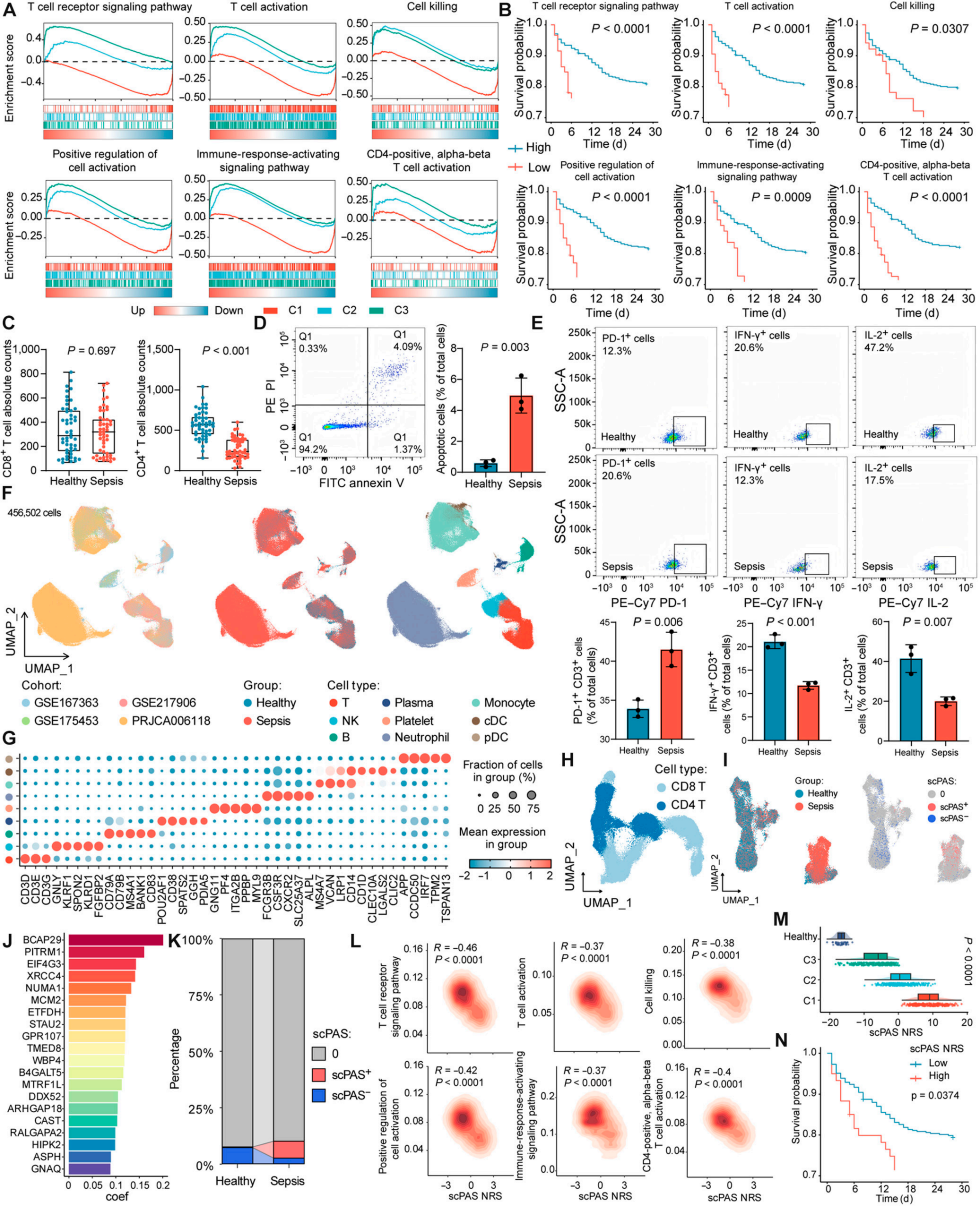
CD4^+^ T cell exhaustion in sepsis patients. (A) Gene set enrichment analysis (GSEA) of 3 distinct clusters. (B) Kaplan–Meier survival analysis of immune-related pathways in sepsis patient prognosis. (C) Absolute counts of peripheral blood CD4^+^ T cells and CD8^+^ T cells in healthy controls (*n* = 50) and sepsis patients (*n* = 50). (D) Flow cytometry analysis of peripheral blood lymphocyte apoptosis in sepsis patients and healthy controls (*n* = 3). (E) Flow cytometry analysis of programmed cell death protein 1 (PD-1), interleukin-2 (IL-2), and interferon-γ (IFN-γ) expression levels in peripheral blood CD4^+^ T cells from sepsis patients and healthy controls. CD4^+^ T cells were isolated from peripheral blood using magnetic beads and activated with anti-CD3/CD28 for 24 h to assess IL-2 and IFN-γ expression levels. Ionomycin, phorbol 12-myristate 13-acetate (PMA), and brefeldin A (BFA) were added 4 h prior to flow cytometry analysis. (F) Integration of single-cell RNA sequencing data and cell annotation landscape from multiple sepsis cohorts. (G) Marker gene expression levels for each cell type. (H) Subdivision of T cells into CD8^+^ T cells and CD4^+^ T cells. (I) Identification of C1-related cells using the scPAS algorithm. (J) Top 20 genes with the highest coefficient values in the logistic regression model fitted by scPAS. (K) Cell proportions of scPAS^+^ and scPAS^−^ cells between groups. (L) Correlation analysis between the normalized risk scores (NRSs) calculated by the scPAS algorithm and immune-related pathways. (M) NRS differences in bulk cohorts. (N) Kaplan–Meier survival analysis of NRS. PE, phycoerythrin; PI, propidium iodide; FITC, fluorescein isothiocyanate; SSC-A, side scatter area; NK, natural killer; cDC, conventional dendritic cell; pDC, plasmacytoid dendritic cell.

We conducted a detailed analysis of the functional differences between the 3 clusters. Gene set enrichment analysis (GSEA) revealed that key immune pathways, including TCR signaling, T cell activation, cell killing, and immune response activation, were significantly suppressed in C1 patients (Fig. 2A and Table S6), while these pathways were notably activated in C3 patients. Notably, the pathway activation in C2 patients exhibited an intermediate phenotype, lying between C1 and C3. Additionally, we performed single-sample gene set enrichment analysis (ssGSEA) to further quantify the activity of these pathways in each patient, followed by Kaplan–Meier survival analysis (Fig. 2B). The results indicated that lower activity of these immune pathways correlated with poorer prognosis. These findings suggest that C1 patients, with the worst prognosis, are in a highly immunosuppressive state, primarily reflecting substantial alterations in T cell function.

To further explore the immune characteristics in sepsis, we analyzed clinical data from 50 patients at the Second Hospital of Hebei Medical University, matched for gender and age with the healthy controls (Table S2). The results revealed that, compared to healthy controls, sepsis patients exhibited a significant increase in neutrophil count and percentage, while lymphocyte count and percentage were markedly reduced (Fig. S1A). Notably, CD4^+^ T cells, rather than CD8^+^ T cells, were significantly decreased in sepsis patients (Fig. 2C), indicating that CD4^+^ T cells play a critical role in adaptive immune suppression during sepsis. We further isolated CD4^+^ T cells from sepsis patients using flow cytometry to analyze their functional impairment. The proportion of apoptotic CD4^+^ T cells was significantly elevated in sepsis patients (Fig. 2D). Moreover, PD-1 expression on CD4^+^ T cells was notably increased (Fig. 2E). After activation with anti-CD3/CD28 antibodies, flow cytometry analysis showed that CD4^+^ T cells from sepsis patients produced significantly less interleukin-2 (IL-2) and interferon (IFN-γ) (Fig. 2E), suggesting that these cells are functionally suppressed and exhausted. Collectively, these results reveal significant changes in the number and function of peripheral immune cells in sepsis patients, especially the reduction of CD4^+^ T cells. These changes likely contribute to the abnormal immune status observed in sepsis and provide a foundation for immune suppression in the later stages of the disease.

Given the significant changes in CD4^+^ T cell function in C1 patients, we tested whether quantifying this subtype feature could predict patient prognosis and immune status, thereby enhancing its clinical translational potential. We first collected single-cell RNA sequencing (scRNA-seq) data from the PBMCs of 4 independent cohorts. After rigorous quality control, more than 450,000 high-quality cells were included in the analysis, including 11 healthy samples and 60 sepsis samples. Based on classical cell marker genes, we annotated all cells (Fig. 2F and G). Subsequently, we further subdivided T cells into CD4^+^ and CD8^+^ subgroups (Fig. 2H) and conducted detailed analysis on CD4^+^ T cells (Fig. 2I and Fig. S1B). We used the scPAS algorithm to correlate the bulk transcriptome-generated subtypes with scRNA-seq data, identifying cells obviously associated with the C1 patient phenotype. The scPAS algorithm fits a logistic regression model to assign coefficient values to each gene and quantifies each cell’s normalized risk score (NRS), based on which cells are classified (Fig. 2I and J and Fig. S1C). Following the principle that no more than 20% of CD4^+^ T cells were selected by the scPAS algorithm [28], we identified 1,105 scPAS^+^ and 1,443 scPAS^−^ CD4^+^ T cells highly correlated with the C1 phenotype. Importantly, nearly all of the scPAS^+^ cells were derived from sepsis patient samples (Fig. 2K), confirming the high reliability of this association. Furthermore, NRS showed a significant negative correlation with the activity of immune-related pathways (Fig. 2L). Quantifying the NRS for each patient in the bulk dataset revealed that sepsis patients had a markedly higher NRS than healthy individuals. Moreover, C1 patients exhibited the highest NRS, with C2 and C3 patients showing progressively lower scores (Fig. 2M), consistent with our previous findings. Finally, Kaplan–Meier analysis demonstrated that patients with a high NRS had poorer prognoses (Fig. 2N). In summary, this model accurately quantifies the C1 subtype preference in sepsis patients and effectively predicts patient prognosis and immune status.

### Monocyte-derived MMP9 identified by serum proteomics as a critical inducer of CD4^+^ T cell exhaustion

**Fig. 3.**
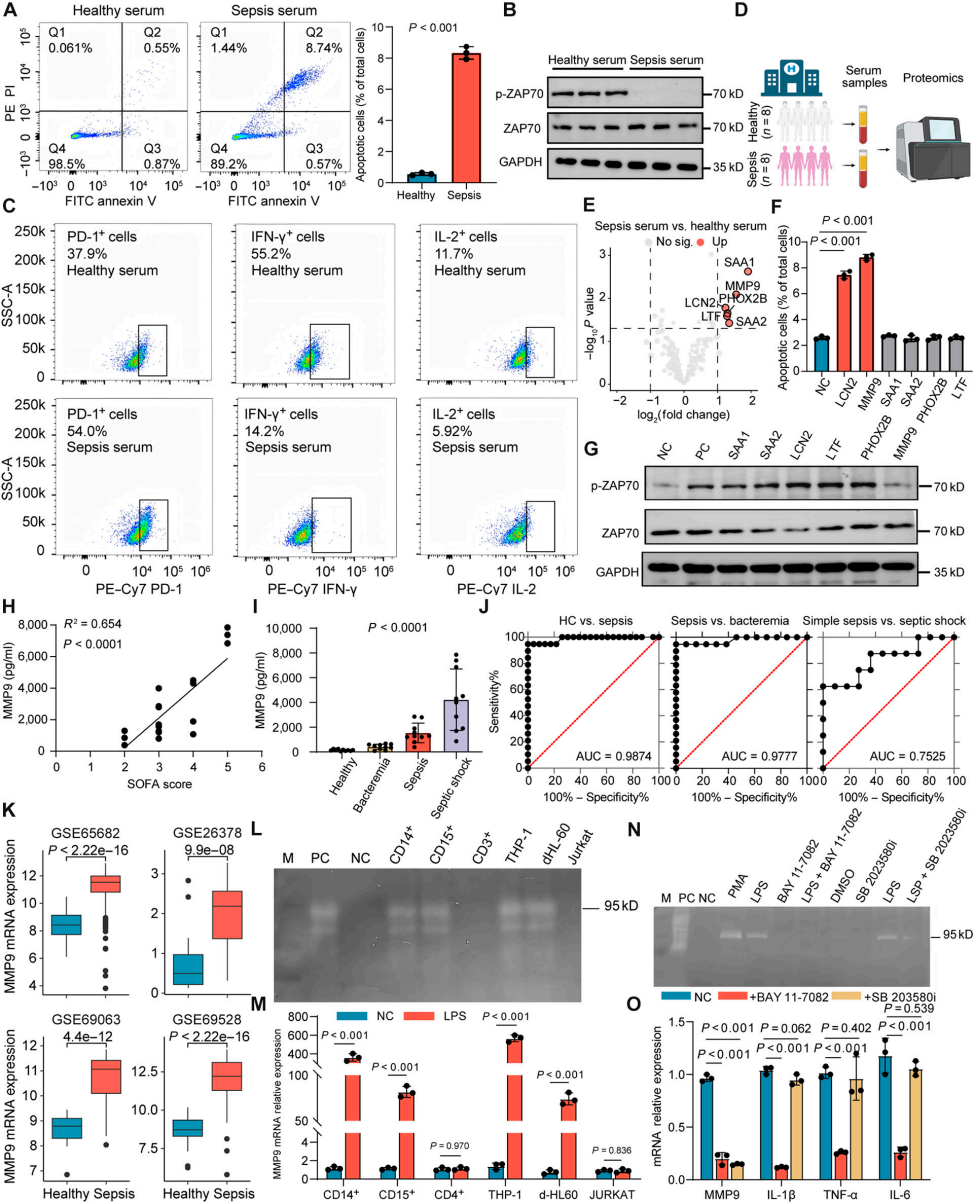
Monocyte-derived matrix metalloproteinase-9 (MMP9) promotes CD4^+^ T cell exhaustion. (A) CD4^+^ T cells were isolated from the peripheral blood of healthy individuals and cultured for 24 h in a medium supplemented with 50% serum from either healthy individuals or sepsis patients. Flow cytometry was used to detect apoptosis rates. (B) Zeta chain of T cell receptor associated protein kinase 70 (ZAP70) phosphorylation levels in human primary CD4^+^ T cells treated with healthy or septic serum. (C) Flow cytometry analysis of PD-1, IL-2, and IFN-γ expression levels in human primary CD4^+^ T cells. (D) Workflow diagram for proteomic sample collection and analysis. (E) Volcano plot showing proteins specifically elevated in sepsis patient serum by proteomic sequencing. (F) Flow cytometry analysis of apoptosis levels induced by 6 recombinant proteins individually. (G) Western blot analysis of ZAP70 phosphorylation levels induced by 6 recombinant proteins individually. (H) Correlation between serum MMP9 levels and sequential organ failure assessment (SOFA) scores in sepsis patients. (I) MMP9 concentrations in clinical samples (*n* = 10). (J) Diagnostic capability of MMP9 in distinguishing healthy controls, simple bacteremia, sepsis patients, and septic shock. (K) MMP9 expression level differences across different bulk cohorts. (L) Gelatin zymography analysis of MMP9 enzyme activity in supernatants from different cell types. PC and NC represent positive and negative controls, respectively. (M) Real-time reverse transcription polymerase chain reaction (RT-qPCR) detection of MMP9 messenger RNA (mRNA) expression levels in different cells following treatment with 1 μg/ml lipopolysaccharide (LPS) or phosphate-buffered saline (PBS) for 24 h. (N) MMP9 enzyme activity in the supernatants of THP-1 cells treated with 1 μg/ml LPS alone, 1 μg/ml LPS + 1 μM BAY 11-7082, 1 μg/ml LPS + 1 μM SB 203580, 100 ng/ml PMA, or dimethyl sulfoxide (DMSO) control for 24 h. (O) Detection of MMP9 and pro-inflammatory cytokine mRNA expression levels. GAPDH, glyceraldehyde-3-phosphate dehydrogenase; LCN2, lipocalin-2; SAA1 and SAA2, serum amyloid A1 and A2; PHOX2B, paired-like homeobox 2B; LTF, lactoferrin; AUC, area under the curve.

Further elucidation of the causes of CD4^+^ T cell exhaustion in high-risk C1 sepsis patients is necessary, as it may be closely associated with disturbances in the immune microenvironment. The interaction between different cell types could shape the unique immune microenvironment of C1 patients. To investigate this, we co-cultured CD4^+^ T cells from the peripheral blood of simple bacteremia patients with sepsis patient serum or healthy control serum. Results showed that, compared to healthy controls, CD4^+^ T cells co-cultured with sepsis patient serum exhibited significantly increased apoptosis rates (Fig. 3A). Additionally, the total protein levels of zeta chain of T cell receptor associated protein kinase 70 (ZAP70), a marker of TCR signaling, did not show significant changes, but ZAP70 phosphorylation levels were markedly reduced (Fig. 3B and Fig. S2A). Simultaneously, PD-1 expression was significantly upregulated, and IL-2 and IFN-γ secretion levels were significantly decreased (Fig. 3C and Fig. S2A), suggesting that certain components in sepsis patient serum may promote the functional exhaustion of CD4^+^ T cells.

To identify key stimulatory proteins, we collected serum samples from patients upon admission for various conditions and monitored their clinical progression. When sepsis developed due to surgery or invasive procedures, additional serum samples were obtained within 24 h of diagnosis. Ultimately, 8 patients were included in the study. By analyzing paired serum samples from the same patients before and after sepsis onset (Fig. 3D), individual variation was minimized. Principal component analysis revealed a clear distinction in serum protein (Fig. S2B). Differential analysis identified 6 proteins that were significantly upregulated in sepsis serum samples, including MMP9, lipocalin-2 (LCN2), serum amyloid A1 (SAA1), serum amyloid A2 (SAA2), paired-like homeobox 2B (PHOX2B), and lactoferrin (LTF) (|log_2_FC| > 1, *P* < 0.05) (Fig. 3E). Notably, no significant expression differences were found between sepsis caused by Gram-negative and Gram-positive bacteria (Fig. S2C). Subsequently, we confirmed this in a larger clinical sample cohort (Table S2 and Fig. S2D). Co-culturing these proteins with primary CD4^+^ T cells for 24 h revealed that LCN2 and MMP9 both significantly increased apoptosis rates in Jurkat cells (Fig. 3F and Fig. S2E). However, only MMP9 significantly inhibited ZAP70 phosphorylation (Fig. 3G and Fig. S2F), suggesting its critical role in regulating T cell quantity and functional abnormalities. Additionally, MMP9 induced increased PD-1 expression on CD4^+^ T cells and reduced IL-2 and IFN-γ production upon anti-CD3/CD28 stimulation (Fig. S2G), confirming the association between MMP9 and CD4^+^ T cell exhaustion during sepsis.

In sepsis patients, serum MMP9 levels were positively correlated with sequential organ failure assessment scores (Fig. 3H). MMP9 levels in the serum of sepsis or septic shock patients were significantly higher than those in simple bacteremia patients and healthy individuals (Fig. 3I), with patient information provided in Table S3. Furthermore, MMP9 levels in septic shock patients were significantly higher than those in simple sepsis patients (Fig. 3I), indicating that MMP9 protein levels are sepsis specific and correlated with disease severity. MMP9 demonstrated excellent diagnostic performance, with an area under the curve (AUC) of 0.9874 for distinguishing sepsis from nonsepsis patients (Fig. 3J), and also showed good performance in distinguishing sepsis from simple bacteremia (AUC = 0.9777) (Fig. 3J). Moreover, MMP9 showed strong performance in distinguishing septic shock from simple sepsis (AUC = 0.7525) (Fig. 3J). These results suggest that MMP9 is closely related to disease severity and has significant diagnostic value. Bulk transcriptome analysis from 10 cohorts showed that MMP9 expression levels were significantly elevated in sepsis samples compared to those in healthy controls, peaking in C1 samples (Fig. 3K and Fig. S2H and I).

However, as PBMCs consist of a mixture of various cell types, including lymphocytes, monocytes, and potentially neutrophils, we isolated CD4^+^ T cells, CD14^+^ monocytes, and CD15^+^ neutrophils from the peripheral blood of healthy individuals. Upon lipopolysaccharide (LPS) stimulation, MMP9 was found to be significantly upregulated in monocytes and neutrophils, but not in lymphocytes (Fig. 3L and M). LPS typically activates the MyD88-dependent signaling pathway through Toll-like receptor 4 (TLR4), inducing the release of acute inflammatory factors [29]. Unlike MMP9, pro-inflammatory cytokines such as IL-1β, IL-6, IL-8, and TNF-α were significantly upregulated in CD14^+^ cells after 4 h of LPS stimulation, while MMP9 expression increased only after 24 h of stimulation (Fig. S2J). This time discrepancy suggests that MMP9 expression is not a direct result of LPS activation but may be a secondary event. Further investigation into the 2 major downstream branches of the MyD88 pathway, nuclear factor-κB (NF-κB) [30] and mitogen-activated protein kinase (MAPK), revealed that treatment of monocytes with the NF-κB inhibitor BAY 11-7082 and the MAPK inhibitor SB 203580 showed that BAY 11-7082 significantly inhibited the production of multiple acute inflammatory factors (including MMP9), while SB 203580 specifically inhibited MMP9 production without affecting other factors (Fig. 3N and O). This delayed expression of MMP9 suggests that its expression may be induced through a positive feedback mechanism involving inflammatory factors in the MAPK pathway.

### Loss of NFAT activity orchestrates the exhaustion program in CD4^+^ T cells during sepsis

**Fig. 4.**
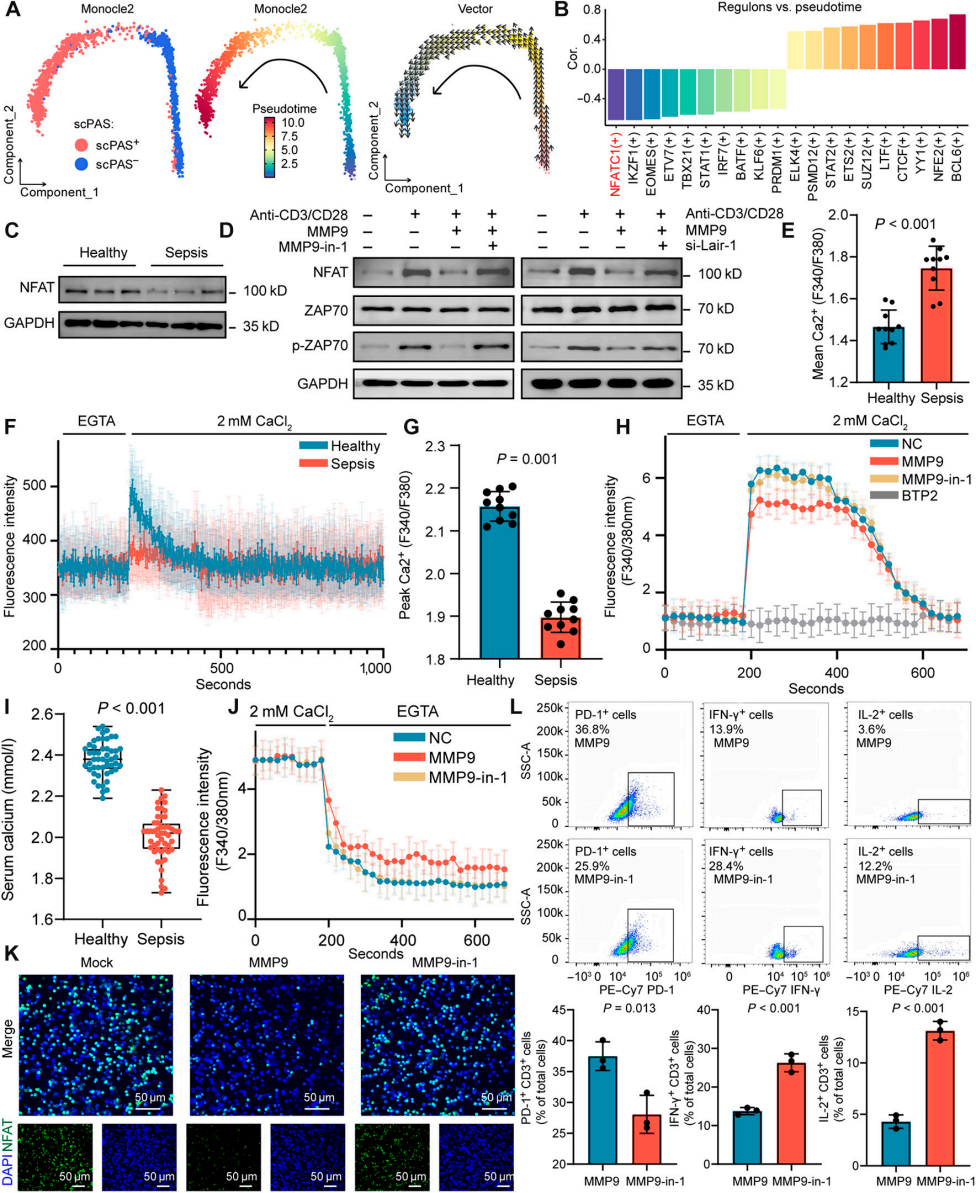
MMP9 promotes calcium ion flow disruption. (A) Pseudotime trajectory analysis of scPAS^+^ and scPAS^−^ cells. (B) Top 10 transcription factors with the highest positive and negative correlations with pseudotime based on transcription factor activity. (C) Nuclear factor of activated T cells (NFAT) expression levels in healthy controls (*n* = 3) and sepsis patients (*n* = 3). (D) Western blot analysis of NFAT, ZAP70, and p-ZAP70 protein levels in Jurkat cells. (E) Analysis of intracellular basal calcium levels in CD4^+^ T cells from healthy controls (*n* = 10) and sepsis patients (*n* = 10) using the Fura-2 fluorescence ratio method. (F) CD4^+^ T cells isolated from healthy individuals or sepsis patients were labeled with 1 μM Fluo-4 AM and suspended in calcium-free extracellular buffer. After treatment with 10 μM thapsigargin (TG) for 10 min, the baseline calcium flux was recorded by flow cytometry. Subsequently, cells were centrifuged at 1,000 rpm for 5 min and resuspended in 2 mM CaCl_2_ buffer, and calcium flux was recorded using flow cytometry. (G) Intracellular calcium flux peaks in CD4^+^ T cells isolated from the peripheral blood of healthy individuals (*n* = 10) or sepsis patients (*n* = 10) following induction with 5 μg/ml anti-CD3/CD28. (H) Jurkat cells were treated with 10 ng/ml MMP9 alone or in combination with 20 μM MMP9-in-1 for 24 h. Cells were labeled with 1 μM Fura-2 AM, suspended in calcium-free extracellular buffer, and treated with 10 μM TG for 10 min. Cells were distributed into black 96-well plates with 3 wells per group. Fluorescence values at 340/380 nm were recorded using a microplate reader for the first 200 s. Subsequently, 2 mM CaCl_2_ buffer was added via an automatic dispensing system, and fluorescence values were continuously recorded for over 600 s; 5 nM BTP2 (a Ca^2+^-release-activated Ca^2+^ [CRAC] channel blocker) was used as a control. (I) Analysis of serum calcium levels in healthy controls (*n* = 50) and sepsis patients (*n* = 50) from clinical data. (J) Jurkat cells were treated with MMP9 alone or in combination with MMP9-in-1 for 24 h, labeled with 1 μM Fura-2 AM, and suspended in 2 mM CaCl_2_ buffer. Following ionomycin treatment for 10 min, cells were dispensed into black 96-well plates with 3 wells per group. Fluorescence values at 340/380 nm were recorded for the first 200 s. Subsequently, 100 μM adenosine triphosphate (ATP) and 2 mM EGTA buffer were added via the automatic dispensing system, and fluorescence values were continuously recorded for over 600 s. (K) Subcellular localization of NFAT. Nuclei were labeled with 4′,6-diamidino-2-phenylindole (DAPI), and NFAT was labeled with FITC. Images were captured under confocal microscopy (×20). All treatment groups were activated with anti-CD3/CD28 for 30 min prior to detection. (L) Flow cytometry detection of PD-1 expression levels in CD4^+^ T cells and IL-2 and IFN-γ expression levels following anti-CD3/CD28 stimulation after MMP9 or MMP9-in-1 treatment. Lair-1, leukocyte-associated immunoglobulin-like receptor-1.

During disease progression, T cells often undergo functional diversification, acquiring specific phenotypes driven by key transcription factors. To delineate this process, we performed pseudotime analysis on scPAS^+^ and scPAS^−^ CD4^+^ T cells based on previous single-cell transcriptomic datasets. Utilizing the Monocle2 algorithm [31], we successfully reconstructed a developmental trajectory from scPAS^−^ to scPAS^+^ subsets (Fig. 4A), a result further supported by the Vector algorithm (Fig. 4A) [32]. Along this differentiation axis, pathways related to T cell activation and immune response were progressively down-regulated (Fig. S3A), consistent with previous findings.

To dissect the underlying regulatory programs, we applied SCENIC to quantify the activity of 368 transcription factors and examined their correlation with pseudotime progression (Fig. 4B). Transcription factors promoting T cell activation, including NFATc1, EOMES, STAT1, IRF7, and KLF6, showed strong negative correlations with differentiation, whereas factors known to promote apoptosis or suppress T cell activation, such as BCL6, SUZ12, and YY1, were positively correlated. Notably, NFATc1 emerged as the most significantly down-regulated factor along the exhaustion trajectory. In bulk RNA sequencing cohorts, NFATc1 activity was markedly suppressed in C1 samples, gradually increased in C2 and C3 samples, and peaked in healthy controls—mirroring phenotypic trends and implicating NFATc1 in the immunopathogenesis of C1 patients. Nuclear factor of activated T cells (NFAT) family members (NFATc1 to NFATc4 and NFAT5) are indispensable transcriptional regulators of immune function, mediating inducible gene expression during immune responses. We observed significantly reduced NFAT protein levels in peripheral CD4^+^ T cells from sepsis patients compared to those in healthy donors (Fig. 4C and Fig. S3H).

### MMP9 disrupts calcium homeostasis to suppress NFAT signaling and T cell activation

NFAT nuclear translocation is regulated by calcium release downstream of TCR activation. Upon stimulation, TCR signaling triggers ZAP70 and downstream effectors, leading to inositol triphosphate (IP3) generation [33,34]. IP3 binds to endoplasmic reticulum (ER)-resident IP3 receptors, inducing Ca^2+^ release into the cytosol. STIM1, sensing ER Ca^2+^ depletion, translocates to the plasma membrane and interacts with Orai1 channels, forming the Ca^2+^-release-activated Ca^2+^ (CRAC) complex and enabling sustained extracellular Ca^2+^ influx. This Ca^2+^ influx activates calcineurin, leading to NFAT dephosphorylation and nuclear import, which drives the expression of genes such as *IL2* and *IFNG*, promoting T cell activation [35]. Thus, the core steps of NFAT activation include calcium signaling initiation, calcineurin activation, NFAT dephosphorylation, and nuclear translocation, wherein store-operated calcium entry (SOCE) plays a central role. In the tumor microenvironment, MMP9 degrades type I collagen, releasing fragments that engage the inhibitory receptor leukocyte-associated immunoglobulin-like receptor-1 (Lair-1) on T cells and attenuate TCR signaling [36]. We first confirmed that Lair-1 expression remained unchanged upon TCR activation in Jurkat cells (Fig. S3C), yet MMP9 treatment promoted its clustering on the plasma membrane (Fig. S3D), suggesting that MMP9 facilitates Lair-1 surface mobilization. Upon stimulation with anti-CD3/CD28, NFAT and p-ZAP70 levels were significantly reduced after 24 h of MMP9 exposure (Fig. 4D). This inhibitory effect was reversed by the MMP9 hemopexin domain inhibitor MMP9-in-1 (Fig. S3E and Fig. 4D). Knockdown of *Lair-1* in Jurkat cells mimicked the rescue effect of MMP9-in-1 (Fig. S3F and Fig. 4D), confirming that MMP9 impairs TCR signaling via Lair-1 engagement.

Given that CRAC–NFAT signaling is central to T cell activation, we evaluated calcium influx following TCR stimulation. MMP9-treated Jurkat cells showed significantly attenuated calcium flux compared to controls (Fig. S3G), which was restored by either MMP9 inhibition or *Lair-1* knockdown (Fig. S3G), indicating that MMP9 impairs calcium signaling. Paradoxically, baseline intracellular calcium levels were elevated in CD4^+^ T cells from sepsis patients (Fig. 4E), despite reduced NFAT dephosphorylation (Fig. 4C and Fig. S3H). To resolve this, we investigated CRAC channel function by depleting ER calcium stores with thapsigargin in calcium-free buffer, followed by calcium readdition and continuous imaging (Fig. 4F). Sepsis-derived CD4^+^ T cells exhibited impaired calcium influx and reduced mobilization peaks (Fig. 4G), suggesting CRAC dysfunction.

To directly assess CRAC channel modulation by MMP9, we used a full-spectrum plate reader to monitor calcium dynamics in Jurkat cells. After MMP9 treatment, cells were loaded with BAPTA-AM (an intracellular Ca^2+^ chelator) and treated with nifedipine and thapsigargin. Upon calcium reintroduction, MMP9 significantly reduced SOCE (Fig. 4H), which was reversed by MMP9-in-1, confirming MMP9 as a negative regulator of CRAC-mediated calcium influx. Although serum calcium levels were significantly reduced in sepsis patients (Fig. 4I), calcium supplementation has been associated with increased mortality [37], likely due to disrupted intracellular calcium clearance. Normally, intracellular calcium is buffered by plasma membrane Ca^2+^-ATPase (PMCA) and sarcoendoplasmic reticulum calcium ATPase (SERCA) pumps to prevent cytotoxic overload. MMP9-treated Jurkat cells showed impaired calcium clearance after ionomycin stimulation in calcium-free conditions, an effect mitigated by MMP9-in-1 (Fig. 4J). ATPase activity assays on isolated plasma membrane and ER fractions confirmed MMP9-mediated suppression of PMCA and SERCA function, reversible by MMP9-in-1 (Fig. S3I). Finally, we assessed the functional impact of MMP9-in-1 on NFAT nuclear localization and T cell effector function. As expected, MMP9 blocked NFAT nuclear import, which was restored upon MMP9 inhibition (Fig. 4K and Fig. S3J). MMP9-in-1 treatment also decreased PD-1 expression and enhanced IL-2 and IFN-γ production upon CD3/CD28 stimulation (Fig. 4L), indicating its potential to restore T cell immunity.

### Selective inhibition of MMP9 reverses T cell exhaustion and restores immune function in septic mice

**Fig. 5.**
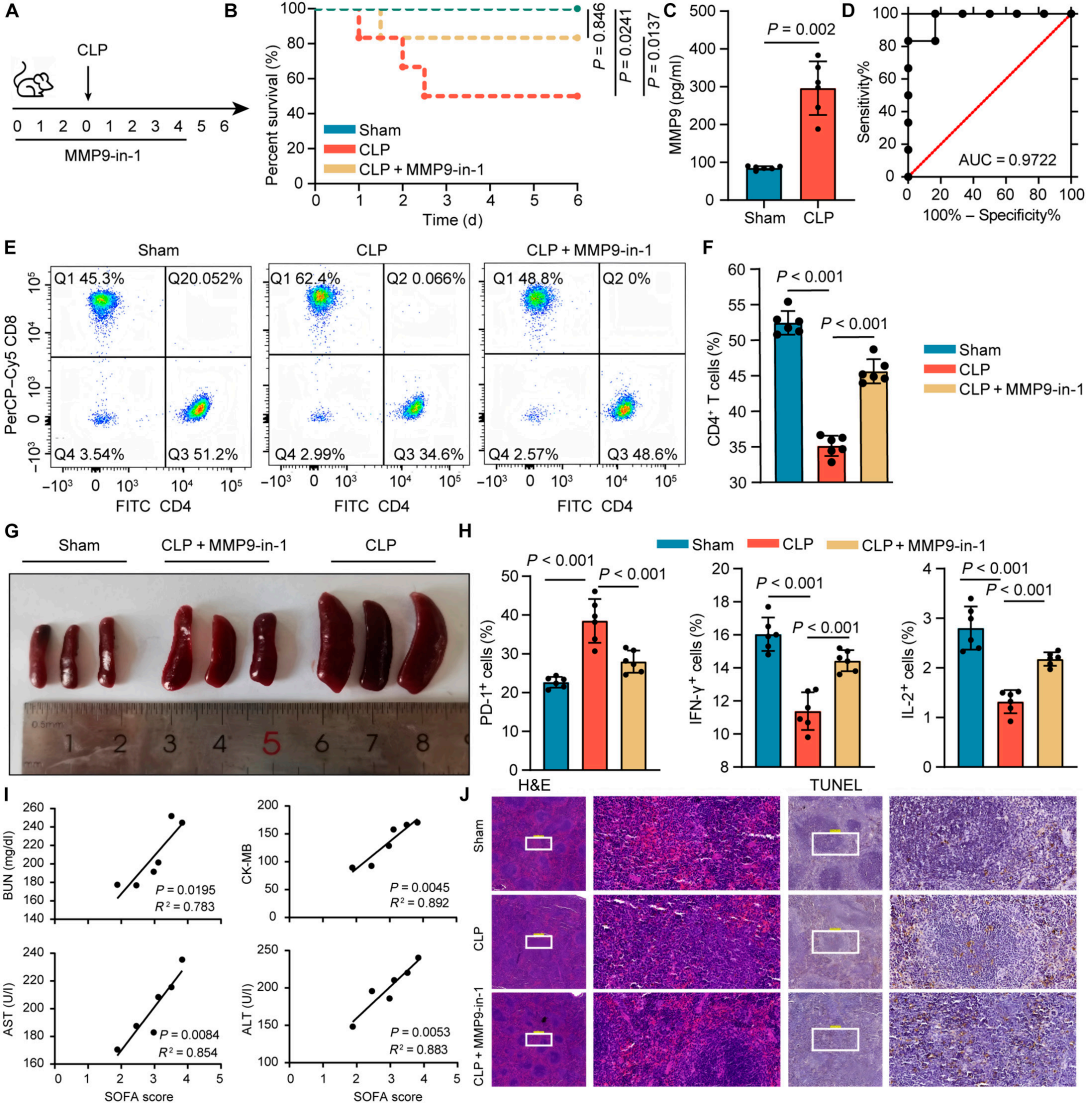
MMP9 inhibition improves prognosis in sepsis mice. (A) Procedures for establishing mouse models. (B) Survival curves of mice in each treatment group. MMP9-in-1: 20 mg/kg body weight. (C) Plasma MMP9 concentrations in the sham group (*n* = 6) and cecal ligation and puncture (CLP) group (*n* = 6) detected by enzyme-linked immunosorbent assay (ELISA). (D) Diagnostic curve of serum MMP9. (E and F) Flow cytometry analysis of CD4^+^ T cell proportions in peripheral blood from various mouse groups (*n* = 6). (G) Spleen index analysis based on spleen size from mouse models in each treatment group (*n* = 3). (H) Flow cytometry measurement of PD-1, IL-2, and IFN-γ expression levels on CD4^+^ T cells from different mouse groups (*n* = 6). (I) Correlation between mouse serum creatinine kinase-MB (CK-MB), alanine aminotransferase (ALT), aspartate aminotransferase (AST), and blood urea nitrogen (BUN) levels with MMP9 levels (*n* = 6). (J) Hematoxylin and eosin (H&E) staining (×5 and ×40) and terminal deoxynucleotidyl transferase dUTP nick-end labeling (TUNEL) staining (×10 and ×40) of pathological sections from mouse spleen. PerCP, peridinin–chlorophyll–protein.

A murine model of sepsis was established via cecal ligation and puncture (CLP), with the experimental design and MMP9-in-1 intervention timeline illustrated in Fig. 5A. Following MMP9-in-1 [38] administration, survival outcomes, pathological changes, and T lymphocyte dynamics were comprehensively evaluated. Treatment with MMP9-in-1 significantly enhanced survival compared to that of the CLP-only group (Fig. 5B). Serum analysis revealed a substantial elevation of MMP9 levels in CLP mice relative to that in sham-operated controls (Fig. 5C). Receiver operating characteristic analysis yielded an AUC of 0.9722, highlighting MMP9 as a robust biomarker for sepsis diagnosis (Fig. 5D). Flow cytometric analysis of peripheral blood revealed a marked reduction in CD4^+^ T cell numbers in CLP mice, which was partially restored following MMP9-in-1 treatment (Fig. 5E and F). These findings implicate MMP9 as a key mediator of CD4^+^ T cell depletion in sepsis and demonstrate that its inhibition confers partial restoration of T cell subsets. Additionally, mice receiving MMP9-in-1 displayed reduced splenomegaly and a significantly decreased spleen index compared to those of the CLP group (Fig. 5G and Fig. S4A). Given the established link between calcium dysregulation and T cell dysfunction, we next assessed calcium homeostasis in CLP mice. Similar to septic patients, CLP mice exhibited a significant decline in serum calcium concentrations and elevated intracellular calcium levels in CD4^+^ T cells. Both abnormalities were notably attenuated by MMP9-in-1 treatment (Fig. S4B). We further investigated the impact of MMP9 inhibition on T cell exhaustion. MMP9-in-1 significantly decreased the expression of the inhibitory receptor PD-1 on CD4^+^ T cells (Fig. 5H and Fig. S4C) and restored IL-2 and IFN-γ production upon stimulation with anti-CD3/CD28 antibodies (Fig. 5H and Fig. S4C). These data confirm that MMP9 inhibition reverses the key features of T cell exhaustion in the septic context. To explore the relationship between MMP9 expression and organ damage, we analyzed tissue injury markers in CLP mice. A positive correlation was observed between MMP9 levels and multiple indicators of tissue pathology (Fig. 5I and Fig. S4D). Histological evaluation via hematoxylin and eosin (H&E) staining revealed widespread inflammatory infiltration and architectural disruption in the spleens of CLP mice, both of which were markedly ameliorated by MMP9-in-1. Terminal deoxynucleotidyl transferase dUTP nick-end labeling (TUNEL) staining further demonstrated extensive apoptosis in CLP spleens, while MMP9-in-1 treatment significantly reduced apoptotic cell numbers (Fig. 5J and Fig. S4E). Furthermore, we evaluated the therapeutic safety of MMP9-in-1. Compared with those in the blank control and solvent control groups, no differences were observed in the levels of organ injury markers in mice from the treatment dose group, which was further confirmed by H&E staining of tissue sections (Fig. S4F and G). In the triple-dose group, a mild elevation in alanine aminotransferase was detected, while the levels of other organ injury markers remained unchanged. H&E staining of the liver indicated mild hepatocellular injury, with no abnormalities observed in other tissues. Collectively, these results provide compelling evidence that MMP9 inhibition mitigates T cell exhaustion and tissue injury, thereby improving immune homeostasis and survival outcomes in septic mice.

## Discussion

Sepsis-induced immunosuppression remains a principal contributor to late mortality; however, the molecular underpinnings of T cell exhaustion in this context are incompletely understood. Our team previously identified sepsis biomarkers (e.g., LILRA5 [18] and monocyte differentiation signatures [19]) via single-omics analysis, focusing on static phenotype association but lacking multi-omics integration, mechanistic validation, and therapeutic exploration. In this study, we identified 3 distinct molecular subtypes of sepsis patients through integrated clustering analyses across large cohorts and multi-omics data. Among them, CD4^+^ T cell exhaustion emerged as a key determinant of poor prognosis in the C1 subtype. Notably, the predictive model we constructed enables early identification of high-risk patients, laying the foundation for rapid clinical translation. Our findings establish MMP9 as a critical regulator of CD4^+^ T cell dysfunction in sepsis, acting primarily through disruption of TCR signaling and intracellular calcium homeostasis. These insights are aligned with emerging data that position proteolytic enzymes as central mediators of immune dysregulation in critical illness.

Although previous studies have implicated MMP9 in extracellular matrix remodeling and leukocyte migration during inflammation and cancer progression [39–42], its direct immunosuppressive function in T cells has received limited attention. Mechanistically, we demonstrate that MMP9 suppresses NFAT activation by impairing TCR signaling via Lair-1-mediated ZAP70 inhibition and by disrupting CRAC-channel-dependent calcium influx—interactions previously unrecognized in the context of sepsis. This dual mechanism aligns with recent reports linking calcium dysregulation to T cell exhaustion in chronic viral infections [43], suggesting the existence of conserved immunopathological pathways across disease states.

Furthermore, reduced ZAP70 phosphorylation emerged as a hallmark of T cell exhaustion in sepsis, echoing findings in cancer and autoimmunity where defective TCR signaling contributes to immunoparalysis [11,44,45]. However, our study uniquely identifies MMP9—not chronic antigen exposure or checkpoint ligands—as an upstream driver of this impairment. The positive correlation between elevated serum MMP9 levels and disease severity reinforces its pathogenic role and resonates with reports linking MMP9 to adverse outcomes in acute respiratory distress syndrome and COVID-19 [46,47]. Importantly, our mechanistic analysis expands the functional scope of MMP9 beyond extracellular matrix remodeling, revealing its capacity to directly perturb intracellular signaling networks—a paradigm shift in the understanding of its role in immune regulation. In contrast to conventional biomarkers that primarily reflect general inflammation, MMP9 stands out by directly mirroring the immunosuppressive phase of sepsis. Its unique ability to induce T cell exhaustion via disrupting TCR signaling and calcium homeostasis positions MMP9 not only as a superior diagnostic and prognostic biomarker but also as a promising therapeutic target for immunoadjuvant therapy.

The therapeutic potential of MMP9 inhibition, demonstrated in our murine models, stands in contrast to the inconsistent outcomes observed with broad-spectrum MMP inhibitors in oncology trials [48]. MMP9-in-1, a selective inhibitor targeting the hemopexin domain, offers a more refined therapeutic approach. The observed restoration of calcium homeostasis and T cell function aligns with recent advances in CRAC-channel-targeted therapies, such as CM4620 [49,50], raising the possibility that combination strategies may synergistically enhance sepsis immunotherapy.

While our study identifies a novel role for MMP9 in sepsis-associated T cell exhaustion, several limitations must be considered. The mechanistic insights are derived primarily from the CLP mouse model and in vitro T cell cultures. Although the CLP model is a standard for polymicrobial sepsis, it may not fully capture the substantial heterogeneity of human sepsis, which is influenced by patient age, comorbidities, pathogen specificity, and infection source. Similarly, reductionist in vitro systems cannot entirely replicate the complex cellular cross talk of the in vivo immune microenvironment. Furthermore, although we observed a correlation between serum MMP9 levels and disease severity, our clinical cohorts were relatively small and geographically homogeneous. Validating these findings in larger, prospective, multicenter cohorts is necessary to establish MMP9 as a robust biomarker across diverse patient populations.

The therapeutic potential of MMP9 inhibition warrants investigation into combination strategies. Since sepsis management relies on timely antibiotics and supportive care, a key direction is to determine whether MMP9-in-1 acts synergistically with standard-of-care antibiotics, potentially addressing both the pathogen burden and the resulting immunosuppression concurrently. Moreover, given that T cell exhaustion is multifactorial, combining MMP9-in-1 with other immunoadjuvants—such as immune checkpoint inhibitors (e.g., anti-PD-1) or cytokines (e.g., IL-7) that target distinct exhaustion pathways—could provide a multilayered approach to more effectively reverse immunoparalysis. Future studies should prioritize evaluating these combination regimens in more complex models and, ultimately, in clinical trials to assess their efficacy and safety.

## Conclusion

**Fig. 6.**
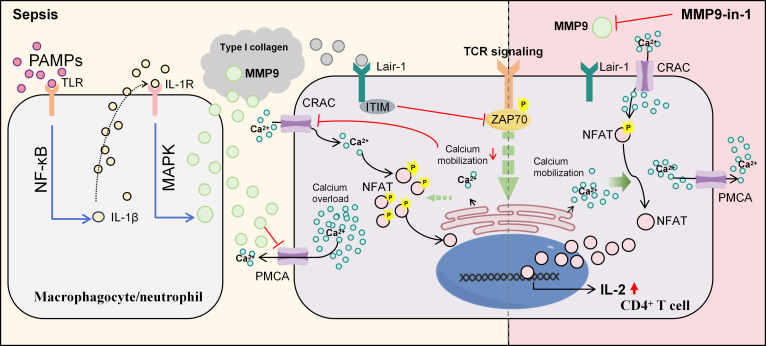
Schematic of the molecular mechanism by which MMP9 drives CD4^+^ T cell dysfunction and exhaustion in sepsis. In response to pathogen-associated molecular patterns (PAMPs) such as LPS, monocytes and neutrophils upregulate MMP9 secretion through Toll-like receptor 4–mitogen-activated protein kinase (TLR4–MAPK) signaling. MMP9 drives CD4^+^ T cell exhaustion via 2 complementary pathways: (a) Binding to Lair-1 on T cells suppresses TCR signaling by inhibiting ZAP70 phosphorylation and endoplasmic reticulum calcium release, thereby attenuating store-operated calcium entry and impairing NFAT dephosphorylation and nuclear translocation, ultimately reducing IL-2 production. (b) Concurrently, MMP9 disrupts calcium homeostasis by inhibiting plasma membrane Ca^2+^-ATPase (PMCA), leading to cytosolic calcium overload and further limiting NFAT activation. The inhibitor MMP9-in-1 reverses these deficits, restoring T cell function and establishing MMP9 as a potential therapeutic target in sepsis-induced immunosuppression. NF-κB, nuclear factor-κB. ITIM, immunoreceptor tyrosine-based inhibitory motif.

This study reveals the molecular network basis of sepsis immune heterogeneity, identifying MMP9 as a key regulator of CD4^+^ T cell exhaustion. Integrating large-scale cohort analysis, single-cell transcriptomics, and functional validation, we established a 3-subtype classification framework where the C1 subtype exhibits the worst prognosis and can be accurately identified through our NRS model. Mechanistically, upon exposure to pathogen-associated molecular patterns, such as LPS, monocytes and neutrophils secrete MMP9 via TLR4–MAPK signaling. MMP9 induces CD4^+^ T cell exhaustion through a dual synergistic mechanism: first, by binding to Lair-1 on T cells, which inhibits ZAP70 phosphorylation and ER calcium release, thereby suppressing SOCE through CRAC channels, and impairing NFAT dephosphorylation and nuclear translocation, leading to reduced IL-2 production; second, by disrupting calcium extrusion via PMCA, resulting in calcium overload and further blocking NFAT activation. The inhibitor MMP9-in-1 reverses these deficits, restoring T cell function (Fig. 6). These findings not only provide a mechanistic rationale for sepsis patient stratification but, more importantly, establish the clinical translational value of MMP9 as an immunotherapeutic target. This MMP9-centered precision inhibitory strategy offers a new therapeutic avenue for overcoming sepsis-induced immunoparalysis.

## Materials and Methods

### Collection and processing of bulk transcriptomic data

We retrieved bulk transcriptomic data from 11 sepsis-related peripheral blood sample (including both whole blood and PBMC) studies available in the Gene Expression Omnibus database, namely, the datasets GSE65682, GSE13904, GSE26378, GSE95233, GSE26440, GSE66890, GSE69528, GSE10474, GSE57065, GSE69063, and GSE32707. A total of 1,862 samples were included in the analysis, comprising 302 healthy samples and 1,560 sepsis samples. For RNA sequencing data, the raw count data were transformed to log_2_(TPM + 1). For microarray data, genes were mapped to symbol format based on the corresponding platform probes. In cases where multiple probes corresponded to the same gene symbol, the average expression level of these probes was taken. Data were normalized using the normalizeBetweenArrays function from the limma package (v3.6). Since each dataset was processed independently, batch effects between datasets were not removed.

### Construction of a molecular interaction disturbance network and unsupervised clustering

Following previous studies [25], we initially constructed a background network of gene interactions using the Reactome database and projected all genes from the expression matrix onto this network. Based on the premise that biological networks are stable and conserved in normal tissues but undergo significant perturbations in sepsis or tumor tissues, we built an interaction disturbance network. The analytical workflow consists of 4 main steps, as illustrated in Fig. 1A. First, for both sepsis and healthy samples, we calculated the rank of each gene within each individual sample based on its expression level, creating a rank matrix. Next, based on the gene interactions within the background network, we applied subtraction along the direction of gene interactions to transform the gene rank matrix into a delta rank matrix. We then used the average delta rank of each gene pair in healthy samples as a reference baseline, comparing it with the delta rank of the corresponding gene pair in sepsis samples. Finally, by subtracting the reference baseline from the delta rank matrix, we obtained the interaction–perturbation matrix, which was subsequently used for unsupervised clustering analysis. Sample clustering was performed using the ConsensusClusterPlus package [51] (v1.66). The maximum number of clusters (*k*) was set to 9, with a step size of 1, to evaluate clustering results across different *k* values. To ensure the robustness of the clustering outcomes, the analysis was repeated 1,000 times. The Pltem parameter was utilized as a feature retention threshold, determining the number of features retained in each bootstrap iteration. In this setup, Pltem was set to 0.8, meaning that 80% of the features were retained in each bootstrap iteration. The clustering process employed the *k*-means algorithm (clusterAlg = “km”) and Euclidean distance (distance = “Euclidean”).

### External extension of subtypes

To perform the external extension of the subtypes, we conducted the NTP analysis [52] (v4.0). NTP is a flexible single-sample prediction method that supports cross-platform and multiclass predictions, along with confidence evaluation. Initially, the top 300 differentially expressed genes for each subtype were identified using the limma method. Subsequently, within external cohorts, the expression profiles of these feature genes were used to identify each subtype. All parameters were set to default values. Visualization of the results was performed using the ComplexHeatmap package [53] (v2.2).

### Functional annotation analysis

Functional annotation analysis was conducted using the ClusterProfiler (v4.12.0) [54] package, incorporating Gene Ontology (GO) [55,56] enrichment analysis, Kyoto Encyclopedia of Genes and Genomes (KEGG) pathway enrichment analysis, GSEA [57], and ssGSEA. Initially, Ensembl gene IDs were converted to standard gene names using the bitr function, followed by GO enrichment analysis via the enrichGO function, covering the biological process, molecular function, and cellular component categories. KEGG pathway analysis was conducted using the enrichKEGG function, with statistical significance defined as *P* < 0.05, and results were corrected for multiple testing using the Benjamini–Hochberg method. For GSEA, the gseGO and gseKEGG functions were used from ClusterProfiler to perform GO and KEGG pathway enrichment analysis. GSEA evaluates the enrichment of predefined gene sets based on the ranking of gene expression levels, using the Kolmogorov–Smirnov test to assess the degree of enrichment. *P* < 0.05 was also applied as the significance threshold, with Benjamini–Hochberg correction. Additionally, ssGSEA was used to evaluate the enrichment score for each sample across specific gene sets, reflecting the activity of each gene set in the sample. This was performed using the ssgsea function from the ClusterProfiler package. All functional enrichment analyses used *P* < 0.05 as the significance threshold, with results adjusted for multiple testing using Benjamini–Hochberg correction.

### scRNA-seq data analysis

Initial data processing was performed using Seurat (v4.0.4), where raw data from 4 sepsis single-cell cohorts were imported using the Read10X function and converted into sparse matrix format. Datasets were integrated using the merge function, with unique cell identifiers ensured by RenameCells. Quality control steps included (a) doublet removal using Scrublet [58,59], (b) filtering of low-quality cells (fewer than 100 genes detected), (c) excluding genes expressed in fewer than 3 cells. Data normalization was performed using LogNormalize scaling (factor = 10,000), and the top 2,000 highly variable genes were identified. Technical variation was regressed out using ScaleData (covariates: unique molecular identifier counts and mitochondrial gene content). Dimensionality reduction was performed using the top 30 principal components, followed by batch effect correction using Harmony [60]. Uniform manifold approximation and projection visualization was applied, and Louvain clustering (resolution = 0.6) was conducted, with the optimal clustering determined via clustree. Cell type annotation was performed using known marker genes and references from the literature.

### Identification of high-risk cells associated with bulk phenotypes from single-cell sequencing data

scPAS [28] (v0.2) is a method that combines bulk transcriptomic data with scRNA-seq data to identify cells highly associated with a specific phenotype based on bulk sample characteristics. In this study, we extracted samples from C1 and C3 patients and classified them into 2 categories, with C1 set to 1 and C3 set to 0. The scPAS function was then used for computation. During this process, we applied an imputation strategy for the expression matrix. Furthermore, to ensure that the selected scPAS^−^ and scPAS^+^ cells did not exceed 20% of the total cell population, we set the alpha parameter to 0.1. While predicting phenotype-associated cells, scPAS fits a logistic regression model for each gene, assigning a coefficient (coef) value, and this model can also be used for external data prediction.

### Pseudotime analysis

Pseudotime analysis was performed using the Monocle2 (v2.32) algorithm [31]. The process began by constructing a cell dataset (CDS) from the raw count data, which was converted into a sparse matrix format suitable for large, sparse datasets typical of scRNA-seq analysis. The CDS was then annotated with phenotype data (e.g., cell type information) and feature data (e.g., gene names), linking the expression data with their biological context. After creating the CDS, size factors were estimated using the estimateSizeFactors function, which normalizes the data to account for sequencing depth differences between cells. Gene dispersion was calculated using estimateDispersions to identify genes with high variability across cells, crucial for distinguishing different cell states. Genes were then detected with the detectGenes function, applying a minimum expression threshold (e.g., at least 3 counts) to filter out low-expressed genes. Genes that contributed most to cell differentiation trajectories were selected based on a significant relationship between their mean expression and dispersion. Gene filtering was based on a predefined dispersion threshold, and the setOrderingFilter function was used to apply the gene selection, focusing the analysis on genes contributing to the ordering of cells along the trajectory. Dimensionality reduction was performed using the reduceDimension function with DDRTree, which efficiently visualizes high-dimensional data and constructs cell trajectories in low-dimensional space. DDRTree identifies the most likely paths through the data, which can then be interpreted as differentiation or developmental trajectories. Cells were subsequently ordered along the trajectory using the orderCells function according to pseudotime.

### Transcription factor activity quantification

Transcription factor activity was assessed using the SCENIC (v1.3.1) approach [61]. Initially, GENIE3 (v1.26.0) was used to construct a regulatory network between transcription factors and their potential target genes based on coexpression patterns. This network was refined by integrating motif relationships and ranking motif–gene regulatory potential, resulting in a refined network called a “regulon”. The regulon includes target genes that are directly bound by transcription factors through upstream motifs. To evaluate the activity of each regulon across all cells, AUCell (v1.26.0) [62,63] was employed.

### Animals

C57BL/6 mice aged 6 to 8 weeks were used in this experiment. Mice were bred and maintained in specific-pathogen-free facilities at Hebei Medical University. All procedures were conducted in accordance with ethical guidelines and animal ethics licensing agreements approved by Hebei Medical University (IACUC-Hebmu-P2022320). The cecum of mice was separated by laparotomy under continuous inhalation anesthesia of 2% isoflurane. The cecum was lapped with 6-0 silk thread at one-half of the cecum and penetrated twice with a 21 puncture needle. The abdominal wall and incision were then closed with 6-0 silk. As a control, mice underwent anesthesia and laparotomy without ligation and cecal puncture. At the appropriate time, blood was collected by cardiac puncture, the mouse was euthanized by excessive inhalation of isoflurane, and the spleen was subsequently removed.

### Source of clinical samples

The study was approved by the Institutional Review Committee of the Second Hospital of Hebei Medical University (2023-R-179). Information on the clinical cohort is provided in the Supplementary Materials. Whole blood was collected from all sepsis patients using heparinized tubes (no. 366667, BD) and serum separator tubes (no. 367987, BD). The PBMCs were isolated from whole blood using a standard Ficoll-Paque isolation method. The CD4^+^ T cells were sorted using magnetic beads (no. 557767, BD), the CD14^+^ cells were sorted using magnetic beads (no. 557769, BD), and the CD15^+^ cells were sorted using Dynabeads CD15 (no. 11137D, Thermo Fisher). Serum was separated by centrifuging at 1,000g for 15 min and stored frozen at −80 °C. The serum for data-independent acquisition (DIA) protein sequencing, or for cell stimulation experiments was inactivated at 56 °C for 30 min and then frozen at −80 °C.

### Clinical cohort

For the DIA proteomic sequencing, the patient’s condition was tracked after hospitalization for various causes, and blood samples were collected once at the initial admission. When the patient developed sepsis due to surgery or invasive operation, blood samples were collected again within 24 h. Blood samples from the same patient before and after the onset of sepsis were used as control and case groups to avoid the influence of individual patient differences and primary disease. The clinical characteristics of this cohort are summarized in Table S1.

For the clinical investigation, we enrolled 100 patients, 50 with and 50 without sepsis, who were matched for sex, age, and primary disease. The diagnosis of sepsis was based on the Third Internationally Agreed Definition of Sepsis and Septic Shock (Sepsis-3). The clinical characteristics of this cohort are summarized in Table S2.

For clinical validation, we included 10 healthy controls, 10 bacteremia patients, 10 sepsis patients, and 10 septic shock patients. The diagnosis of bacteremia was based on positive blood culture before antibiotic application, and a single positive culture vial was excluded. Patients with bacteremia and sepsis were matched by sex, age, and primary disease. The clinical characteristics of this cohort are summarized in Table S3.

### DIA proteomic sequence

Clinical information of samples undergoing proteomic sequencing is shown in Table S1. DIA proteomic sequencing was completed by Shanghai Applied Protein Technology Co., Ltd.

### Cell culture and treatment

Jurkat E6.1 (RRID: CVCL_0065), THP-1 (RRID: CVCL_0006), and HL-60 (RRID: CVCL_0002) cell lines were purchased from Wuhan Pricella Biotechnology Co., Ltd (Wuhan, China). The cells were cultured in RPMI 1640 (no. C11875500BT, Gibco) or Iscove’s modified Dulbecco’s medium (no. C12440500BT, Gibco) suspension cell growth medium supplemented with penicillin–streptomycin solution (100 μg/ml, no. C0222, Beyotime) and 10% or 15% fetal calf serum (no. 10099141C, Gibco). The composition and concentration of all reagents for cell experiments were as follows: 5 μg/ml anti-human CD3/CD28 monoclonal antibody (no. GMP-TL101/102, T&L Biotechnology), 1 μg/ml LPS (no. L2630; Sigma), 1 μM BAY 11-7082 (no. HY-13453), and 1 μM SB 203580i (no. S1076, Selleck). The recombinant proteins used in the experiment are shown in Table S4.

### Flow cytometry

The flow cytometry experiment was conducted on a BD Bioscience flow cytometer. The flow cytometry procedure was as follows: 50 μl of EDTA-anticoagulated whole blood was taken, and 5 μl of the corresponding antibody was added for 30 min of surface staining. For intracellular factor staining, BD Cytofix/Cytoperm Plus (no. 555028) was used to block secretion and fix membrane permeabilization, followed by staining. Details of the antibodies used can be found in the Supplementary Materials (Table S4).

### RT-qPCR and Western blot analysis

For real-time reverse transcription polymerase chain reaction (RT-qPCR) assay, total RNA was extracted from PBMCs according to TRIzol reagent (no. 15596026C, Invitrogen) instructions. The Reverse Transcriptase Kit (no. RR037A, Takara) was used to synthesize complementary DNA, and the TB Green PCR Kit (no. CN830A, Takara) was used for qPCR. The primer sequences are in Table S5.

For Western blot analysis, the total protein of PBMCs was extracted with radioimmunoprecipitation assay buffer (no. P0013B, Beyotime) supplemented with phenylmethylsulfonyl fluoride (no. P1045, Beyotime). Electrophoresis and membrane transfer and blocking with 5% bovine serum albumin (no. 4240GR005, BioFroxx) were performed. Afterward, the membranes were incubated with primary antibodies and then secondary antibodies. The results were detected by the chemiluminescence method. Details of the antibodies used can be found in the Supplementary Materials (Table S4).

### Enzyme-linked immunosorbent assay

Human MMP9 enzyme-linked immunosorbent assay (ELISA) kits (no. abs510010), IL-2 ELISA kits (no. abs510001), mouse MMP9 ELISA kits (no. abs552243), Blood Urea Nitrogen Microplate Assay Kit (no. abs580197), Alanine Transaminase Microplate Assay Kit (no. abs580002), Aspartate Transaminase Microplate Assay Kit (no. abs580004), and mouse CK-MB ELISA kits (no. abs552211) were purchased from Absin and used according to the manufacturer’s procedures.

### MMP9 activity assay

The activation of recombinant human MMP9 (rhMMP9) and the generation of collagen fragments were carried out according to the methods of Vijver et al. [36]. The activity of rhMMP9 was detected by gelatin enzyme spectrometry(no. RTD6173, Real-times).

### siRNA knockdown

For the RNA interference knockdown experiments, a small interfering RNA (siRNA) against human *Lair-1* was designed and synthesized from GenePharma. Cells were transiently transfected with 30 nM siRNA using Lipofectamine 2000 according to the manufacturer’s protocols. The sequences of the siRNA duplex used are in Table S5.

### Intracellular calcium and calcium flux measurement

The cells were resuspended in Hank’s balanced salt solution and loaded with 2 μM Fura 2-AM (no. S1052, Beyotime) in the dark. After washing, the cells were resuspended in calcium-free liquid. The fluorescence intensity at 340/380 nm was detected using a TECAN M1000 Pro fluorescence microplate reader. To measure calcium flux, fluorescence signals were acquired every 20 s over a period of 9 min to monitor calcium dynamics. All inducing or blocking reagents were added via the automatic dispensing system of the microplate reader. For flow cytometry detection, cells were loaded with Fluo-4 AM (no. S1060, Beyotime), washed, and resuspended. After recording a 200-s baseline, an equal volume of 4 mM CaCl_2_ was added to expose the cells to an extracellular calcium environment, and the fluorescence intensity in the FITC channel was continuously recorded for over 10 min.

### Immunohistochemistry and immunofluorescence

For H&E staining, all tissues were subjected to standard paraffin embedding and 5-μm sections were used. TUNEL staining was performed using an in situ TUNEL staining kit (no. C1091, Beyotime).

For the immunofluorescence of cells, cells were adhered to poly-l-lysine-treated coverslips, then permeabilized, blocked, and incubated with primary antibodies and then fluorescent secondary antibody. Coverslips were mounted on the glass slides for confocal imaging (Olympus FV3000). Details of the antibodies used can be found in the Supplementary Materials (Table S4).

### ATPase activity assay

Cells were gently lysed using a lysis buffer containing 0.25 M sucrose and 1 mM EDTA. Initial precipitation was obtained through differential centrifugation. The precipitate was then layered over a 20% to 50% sucrose gradient and centrifuged at 100,000g for 3 h. The plasma membrane was collected from the high-density layer, while the ER membrane was gathered from the low-density layer. ATPase activity was assayed using the malachite green method according to the manufacturer’s instructions (Malachite Green Phosphate Detection Kit, no. S0196S, Beyotime).

### Statistical analysis

Data processing, visualization, and statistical analysis were conducted using the R 4.4.0 software. The correlation between 2 continuous variables was evaluated using Spearman’s rank correlation coefficient. Initially, normality tests were performed on the datasets. For data exhibiting a normal distribution and homogeneity of variance, the Student *t* test and one-way analysis of variance were employed to compare differences between 2 or more groups. In contrast, for nonnormally distributed data or those not meeting the assumption of homogeneity of variance, the Wilcoxon test and Kruskal–Wallis test were used for comparing 2 groups and multiple groups, respectively. Categorical variables were analyzed using the chi-square test. To ensure reliability, the experiment was repeated 3 times to confirm the consistency of the results. The results were considered statistically significant at *P* < 0.05.

## Ethical Approval

All animal experimental procedures were conducted in accordance with ethical guidelines and approved by the Animal Ethics Committee of Hebei Medical University (IACUC-Hebmu-P2022320). All studies involving human clinical samples were approved by the Ethics Review Committee of the Second Hospital of Hebei Medical University (2023-R-179).

## Data Availability

The mass spectrometry proteomics data have been deposited to the ProteomeXchange Consortium (https://proteomecentral.proteomexchange.org) via the iProX partner repository with the dataset identifier PXD055435. The characteristic genes of the 3 subtypes and the gene coefficients of the logistic regression model are stored at https://github.com/AcetylCoALab/Sepsis_data.
