# Supplementary material for: Targeting Matrix Metalloproteinase-9 to Alleviate T Cell Exhaustion and Improve Sepsis Prognosis
Source: Research (Wash D C). 2025 Nov 25;8:0996. doi: 10.34133/research.0996 (PMC12645450; doi:10.34133/research.0996)
Supplement: Supplementary 1 — Figs. S1 to S4 Tables S1 to S6 [file research.0996.f1.zip › Supplementay Materials.docx]

**Supplement figure legends**

**
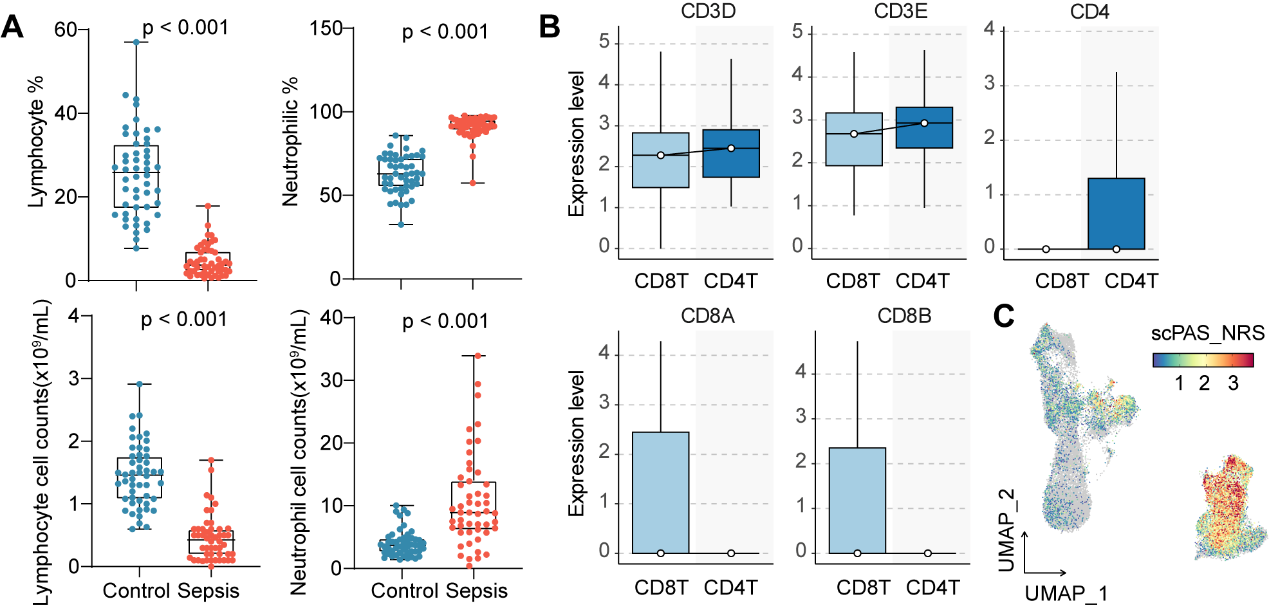
**

**Figure S1. Flow cytometry and single-cell RNA sequencing analysis reveal CD4+ T cell function in sepsis**

**A**: Clinical data analysis of neutrophil percentage and absolute count, lymphocyte percentage and absolute count. **B**: Expression differences of CD3D, CD3E, CD4, CD8A, and CD8B between CD4^+^ T cells and CD8^+^ T cells, demonstrating high reliability of subgroup classification. **C**: NRS values calculated by scPAS analysis.


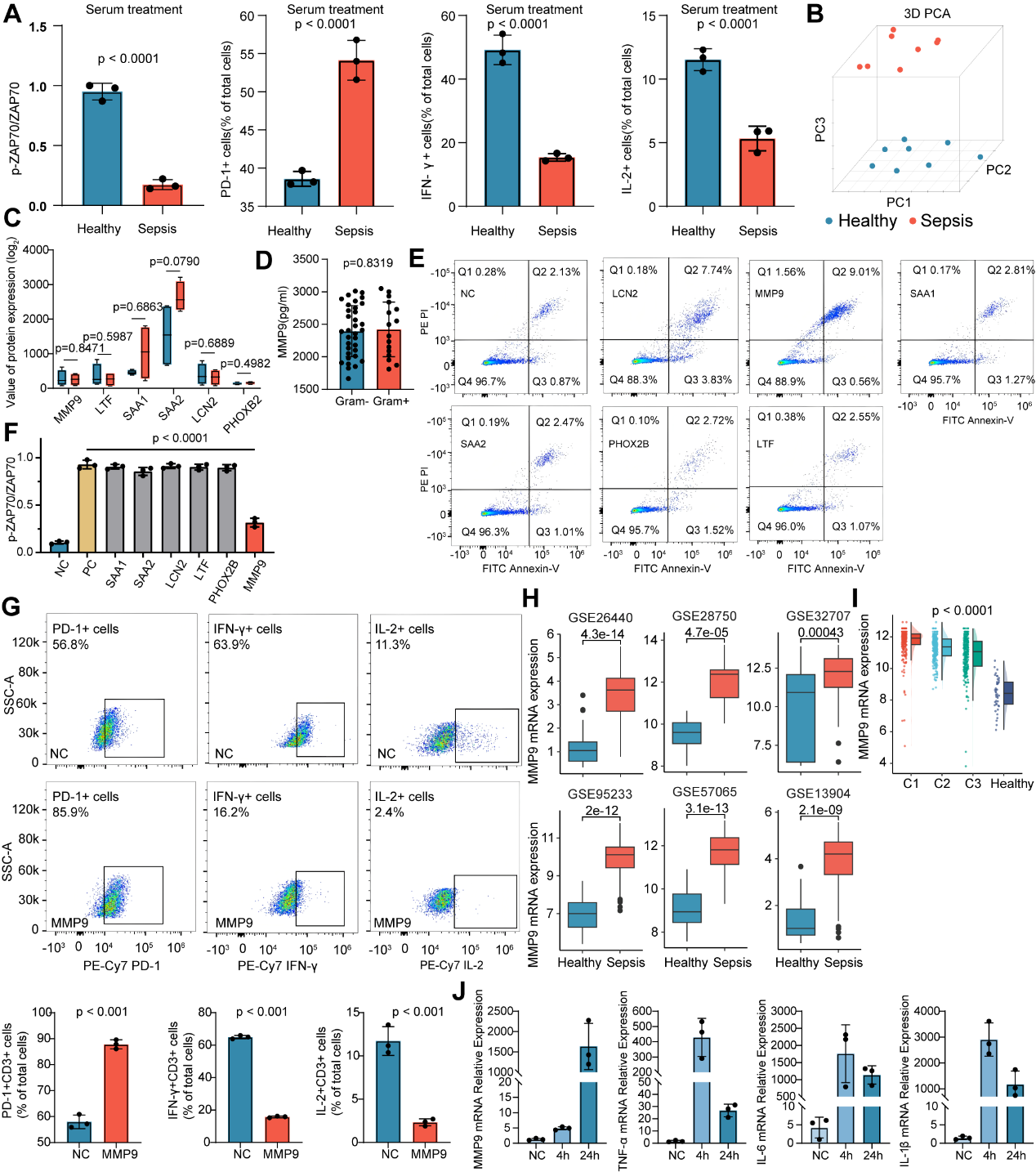


**Figure S2. MMP9 promotes CD4+ T cell apoptosis**

**A**: Statistical analysis of ZAP70 phosphorylation levels and PD-1, IFN-γ, and IL-2 expression levels in human primary CD4^+^ T cells after 24-hour stimulation with healthy or sepsis patient serum (n=3). **B**: Principal component analysis (PCA) plot showing proteins specifically elevated in sepsis patient serum by proteomic sequencing. **C**: Expression levels of elevated proteins in sepsis caused by Gram-negative versus Gram-positive bacteria in proteomic profile analysis. **D**: Expression levels of MMP9 in sepsis caused by Gram-negative versus Gram-positive bacteria. **E**: Flow cytometry detection of cell apoptosis in human primary CD4^+^ T cells after stimulation with six recombinant proteins. **F**: Statistical analysis of ZAP70 phosphorylation levels in human primary CD4^+^ T cells after stimulation with six recombinant proteins (n=3). **G**: Flow cytometry detection of PD-1 expression levels in human primary CD4^+^ T cells and IL-2 and IFN-γ expression levels following anti-CD3/CD28 stimulation after MMP9 recombinant protein treatment (n=3). **H**: MMP9 expression levels across different bulk cohorts. **I**: MMP9 expression levels in different cluster patients. **J**: Expression levels of IL-1β, IL-6, TNF-α, and MMP9 genes in THP-1 cells at 0, 4, and 24 hours after 1 μg/mL LPS treatment (n=3).


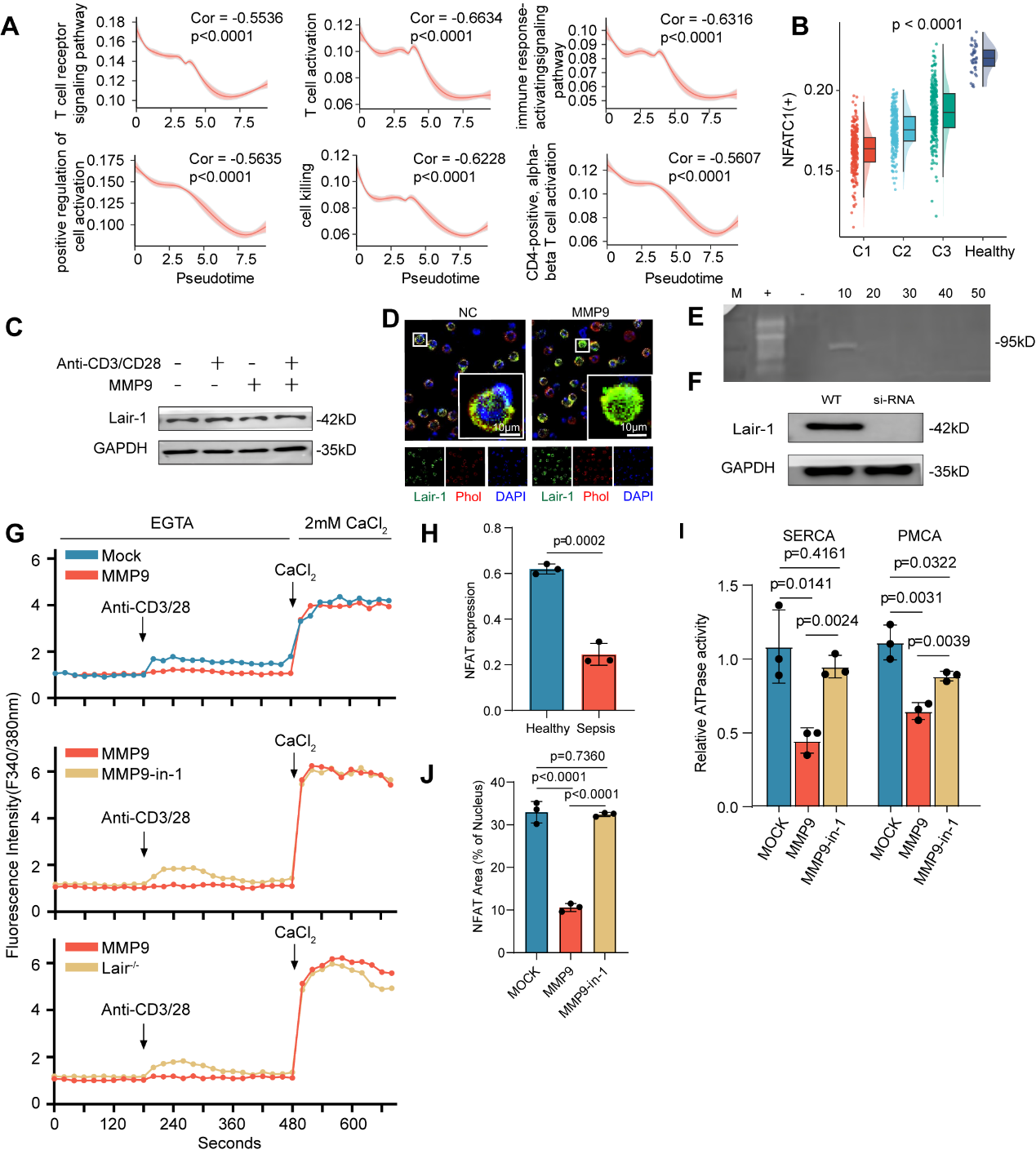


**Figure S3. MMP9 stimulation leads to calcium flow disruption in CD4^+^ T cells**

**A**: Correlation between different immune pathway activities and pseudotime. **B**: NFATC1 activity differences among different cluster patients. **C**: Western blot detection of Lair-1 expression with or without anti-CD3/CD28 and MMP9 treatment. **D**: Immunofluorescence detection of Lair-1 localization and distribution in Jurkat cells. Cytoskeleton was labeled with Phalloidin, nuclei with DAPI, and Lair-1 with FITC. Images were captured under confocal microscopy at 40× magnification. **E**: Validation of MMP9 inhibitor effects. **F**: Western blot verification of Lair-1 siRNA knockdown. **G**: Calcium flux analysis in Jurkat cells under various conditions: induced by 5 μg/mL anti-CD3/CD28 and 2 mM CaCl₂ with or without 10 ng/mL recombinant human MMP9 protein; with MMP9 protein in presence or absence of MMP9-in-1; and with MMP9 protein in Lair-1 knockdown cells. **H**: NFAT expression levels in healthy controls (n=3) and sepsis patients (n=3). **I:** Plasma membrane and endoplasmic reticulum membrane separation from Jurkat cells using differential centrifugation and sucrose density gradient centrifugation, with ATPase activity detection using the Malachite Green method. **J:** Statistical analysis of NFAT subcellular localization.


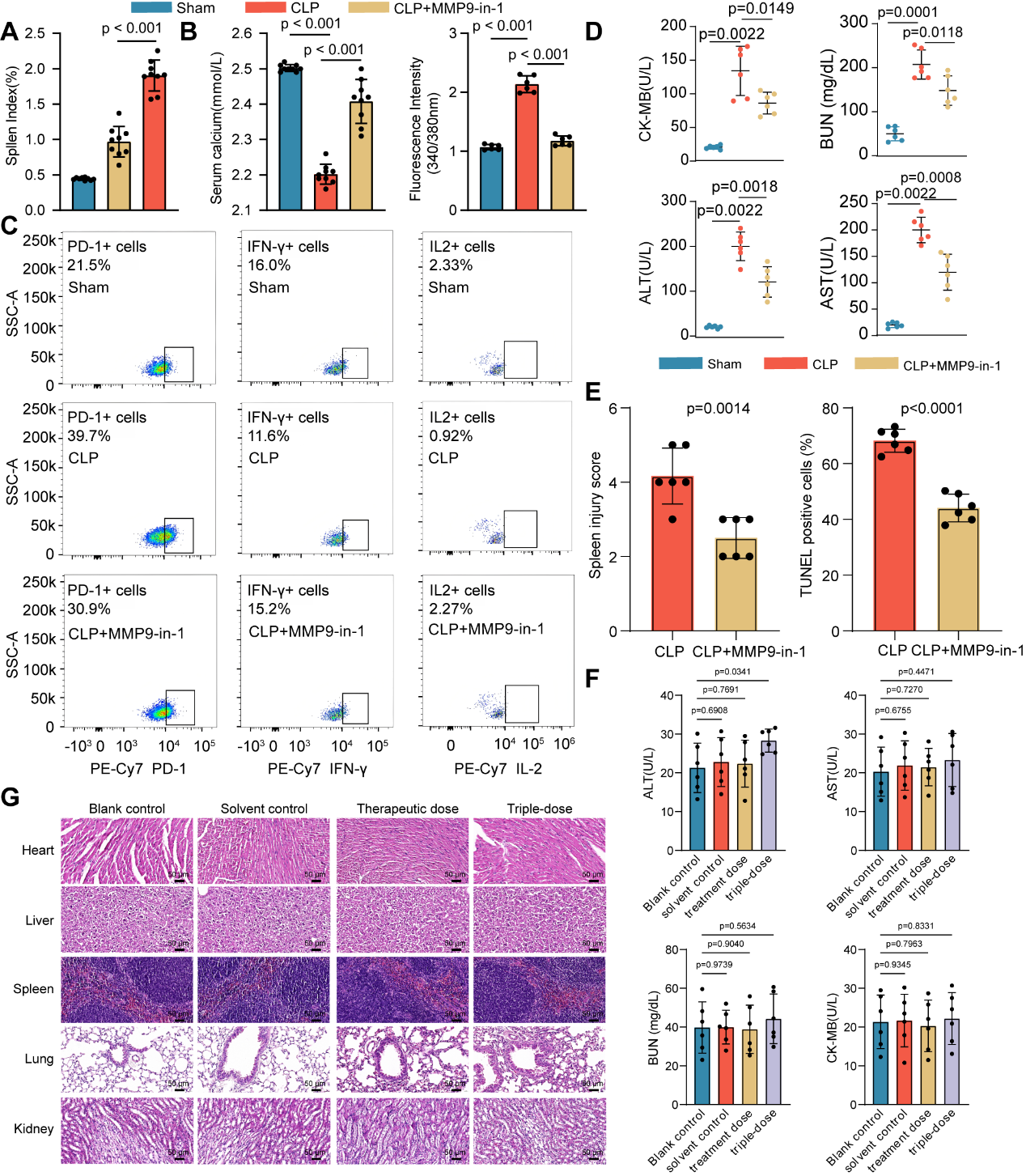


**Figure S4. MMP9 inhibition improves prognosis in sepsis mice**

**A:** Spleen index in sham, CLP, and MMP9-in-1 treatment groups (n=6). **B:** Statistical analysis of serum calcium ion concentrations and intracellular basal calcium levels in sham, CLP, and MMP9-in-1 treatment groups (n=6). **C:** Flow cytometry analysis of PD-1, IFN-γ, and IL-2 expression levels in peripheral blood CD4^+^ T cells from sham, CLP, and MMP9-in-1 treatment groups. **D:** Serum CK-MB, BUN, ALT, and AST levels in sham, CLP, and MMP9-in-1 treatment groups (n=6). **E:** Statistical analysis of spleen injury scores and apoptotic cell proportions in CLP and MMP9-in-1 treatment groups (n=6). **F**: Serum CK-MB, BUN, ALT, and AST levels in Blank Control, Solvent Control, Treatment Dose and Triple-Dose groups. **G**: H&E staining (40×) of heart, liver, spleen, lung, and kidney tissues in mouse from the Blank Control, Vehicle Control, Therapeutic Dose, and Triple-Dose Groups.
